# Warmer Weather Linked to Tick Attack and Emergence of Severe Rickettsioses

**DOI:** 10.1371/journal.pntd.0000338

**Published:** 2008-11-18

**Authors:** Philippe Parola, Cristina Socolovschi, Luc Jeanjean, Idir Bitam, Pierre-Edouard Fournier, Albert Sotto, Pierre Labauge, Didier Raoult

**Affiliations:** 1 Unité de Recherche en Maladies Infectieuses et Tropicales Emergentes, CNRS-IRD UMR 6236, WHO Collaborative Centre for Rickettsial and Other Arthropod Borne Bacterial Diseases, Faculté de Médecine, Marseille, France; 2 Consultation de Neuro-Ophtalmologie, CHU Nîmes, France; 3 Service des Maladies Infectieuses, CHU Nîmes, France; The George Washington University, United States of America

## Abstract

The impact of climate on the vector behaviour of the worldwide dog tick *Rhipicephalus sanguineus* is a cause of concern. This tick is a vector for life-threatening organisms including *Rickettsia rickettsii*, the agent of Rocky Mountain spotted fever, *R. conorii*, the agent of Mediterranean spotted fever, and the ubiquitous emerging pathogen *R. massiliae*. A focus of spotted fever was investigated in France in May 2007. Blood and tissue samples from two patients were tested. An entomological survey was organised with the study of climatic conditions. An experimental model was designed to test the affinity of *Rh. sanguineus* for biting humans in variable temperature conditions. Serological and/or molecular tools confirmed that one patient was infected by *R. conorii*, whereas the other was infected by *R. massiliae*. Dense populations of *Rh. sanguineus* were found. They were infected with new genotypes of clonal populations of either *R. conorii* (24/133; 18%) or *R. massiliae* (13/133; 10%). April 2007 was the warmest since 1950, with summer-like temperatures. We show herein that the human affinity of *Rh. sanguineus* was increased in warmer temperatures. In addition to the originality of theses cases (ophthalmic involvements, the second reported case of *R. massiliae* infection), we provide evidence that this cluster of cases was related to a warming-mediated increase in the aggressiveness of *Rh. sanguineus*, leading to increased human attacks. From a global perspective, we predict that as a result of globalisation and warming, more pathogens transmitted by the brown dog tick may emerge in the future.

## Author Summary

The impact of climate on the behaviour of the worldwide dog tick *Rhipicephalus sanguineus* is a cause of concern. This tick is a vector for life-threatening organisms including *Rickettsia rickettsii*, the agent of Rocky Mountain spotted fever, *R. conorii*, the agent of Mediterranean spotted fever, and the ubiquitous emerging pathogen *R. massiliae*. A focus of spotted fever was investigated in France in May 2007. One patient was found to be infected by *R. conorii*, whereas the other was infected by *R. massiliae*. Theses cases were original because of ophthalmic involvements, and the report of the second case of *R. massiliae* infection in the scientific literature. During an entomological survey, dense populations of *Rh. sanguineus* were found in the house where the patient had been bitten by ticks. Ticks were infected with either *R. conorii* or *R. massiliae*. Interestingly, April 2007 was the warmest since 1950, with summer-like temperatures. In this work, we show that the human affinity of *Rh. sanguineus* is increased in warmer temperatures, and provide evidence that this cluster of cases was related to a warming-mediated increase in the aggressiveness of *Rh. sanguineus*, leading to increased human attacks. From a global perspective, we predict that as a result of globalisation and warming, more pathogens transmitted by the brown dog tick may emerge in the future.

## Introduction

The recent outbreak of Chikungunya virus in the Indian Ocean Islands and India [1] that reached Europe in 2007 [2], has illustrated the current medical importance of globalising vector-transmitted infections [3]. The impact of ticks on human public health was recognised with the emergence of Lyme disease 25 years ago [4]. Since then, around 15 emerging tick-borne rickettsioses have emerged [5].

Ticks strive for the best conditions for their life cycle and to find and bite a host [6]. Climate and the availability of hosts are among the major factors influencing ticks [6],[7]. The impact of climate change on tick-borne diseases has been the topic of controversial debate in the scientific literature [7],[8]. Additionally, it has been suggested that global warming would result in a northward expansion of several tick species, including *I. ricinus* and *I. scapularis* the vectors of Lyme disease in Europe and North America, respectively [7],[9], and *Rhipicephalus sanguineus* group ticks [10].

*Rh. sanguineus*, the dog brown tick, is considered the first globalised tick as it has become the most widespread tick throughout the world due to its specialised feeding on the domestic dog [11],[12]. In addition to the dog pathogens *Babesia canis* and *Ehrlichia canis* [12], *Rh. sanguineus* is known to transmit two life threatening rickettsial diseases in humans, Mediterranean spotted fever (MSF) caused by *R. conorii* in the old world [13], and Rocky Mountain spotted fever (RMSF) caused by *R. rickettsii* in America [14]. *Rh. sanguineus* also transmits *R. massiliae*, a worldwide emerging pathogen [15] with a single documented case of infection [16].

*Rh. sanguineus* is well adapted to urban environments and lives in close contact with humans. However, *Rh. sanguineus* rarely feeds on humans, particularly in temperate countries [11]. Herein, we describe the investigation of a focus of rickettsioses in southern France during the exceptionally warm April and May months of 2007. Patients suffered from severe *R. conorii* and *R. massiliae* infections, and we found that this cluster of cases resulted from unexpected proliferation and aggressive behaviour of *Rh. sanguineus* ticks infected by these rickettsiae. We demonstrate experimentally that *Rh. sanguineus* readily bites humans when exposed to higher temperatures.

## Methods

### Case reports

#### Patient 1

**Figure 1:**
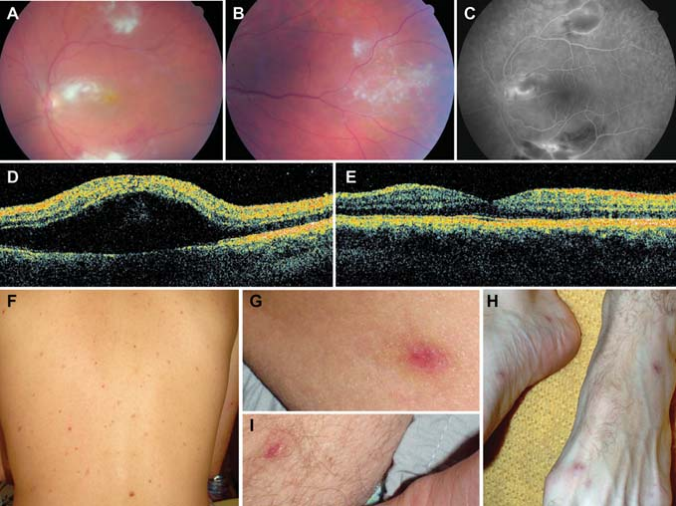
Chorioretinitis with macular involvement in patient 1 with *Rickettsia massiliae* infection. Fundus examination shows numerous intraretinal haemorrhages around three white medium-sized lesions including one located near the macula. An important macular oedema was observed in the left eye (Panel A), and a mild inflammation with a white medium-sized retinal lesion was present in the right eye (Panel B). Panel C shows a fluorescein angiography with retinal vascular leakage around hypofluorescent lesions in the right eye. Optical Coherence Tomography showed an important macular oedema on the left eye (panel D) compared to the right eye (panel E). Panel F shows a maculopapular rash still present three weeks after the onset of ophthalmic symptoms. Inoculation eschars were still visible on the trunk (Panel G and H) and on the feet (Panel I).

On May 24^th^, 2007, a 25-year-old man developed fever, night sweats, headache, two skin necrotic lesions on the buttocks and the thighs, respectively, and a maculopapular rash. On June 10^th^ he complained of acute visual loss. A bilateral chorioretinitis was diagnosed (**Figure 1**). A maculopapular rash was still present involving the palms and soles (**Figure 1**). He was initially treated with intravenous ganciclovir as well as eye drops as a cytomegalovirus infection was suspected. On June 23^rd^ (day 29), a late serum tested positive for spotted fever group rickettsiae. Oral doxycycline plus ofloxacin was started with an intravenous methylprednisolone regimen. The clinical course was favourable, but three months later the recovery of visual acuity was incomplete and fundus examination of the left eye showed a disorganisation of the macular neuroretina. When interviewed again, the patient reported contact with ticks when he visited a friend at the beginning of May. Written informed consent has been obtained from this patient to report case details and pictures.

#### Patient 2

A previously healthy 30-year-old man (a friend of patient 1) developed high fever, night sweats and headache in May 2007 followed by a maculopapular rash involving the palms 10 days later. A cicatricial inoculation eschar was found on the skin of the left axilla. He presented with acute bilateral visual loss associated with confusion, loss of hearing and tinnitus. A bilateral chorioretinis was diagnosed. An empiric doxycycline plus ofloxacin treatment was started and he recovered. He reported contact with ticks when he visited the same friend as patient 1. Written informed consent has been obtained from this patient to report case details.

### Laboratory testing

Serum, spinal fluid, aqueous humor and tissue specimens were obtained from both patients. Sera were tested for IgG and IgM antibodies by immunofluorescence (IF) assay [17] using 10 rickettsial antigens [18]. When cross-reactivity was noted between several antigens, and the difference in titers between antigens was lower than two-fold, western blotting following cross-adsorption was performed [17]. DNA was extracted from ground eschar biopsies and aqueous humor specimens from patient 1 and from acute sera from both patients [19]. These extracts were used as templates in a nested PCR assay incorporating primers selected from specific regions of the *pgsA* gene present in both *R. conorii* and *R. massiliae* genomes (GenBank accession numbers NC 003103 and CP 000683, respectively). Primers specifically designed for this study included *pgs*AF1 (5′- AGATAATGTAGATGAGATACC-3′), *pgs*AR1 (5′-GTTAAAAAAGCGGCAATCCA-3′), *pgs*A F2 (5′-TTTTTAGTTAGCGGTCTTCGG-3′) and *pgs*AR2 (5′- TTGAGCCTAGTATCAATATCG-3′). The so-called “suicide PCR” procedure has been followed, that is a nested PCR using single-use primers targeting a gene never amplified previously in the laboratory. This procedure avoids “vertical” contamination by amplicons from previous assays, one of the limitations of extensive use of PCR [20]. Sequences of positive PCR products were compared to GenBank [21].

### Detection of *Rickettsia spp.* in ticks by PCR and Multi-spacer typing (MST) genotyping

Tick DNA was extracted and rickettsiae were detected in each sample through the PCR amplification of a 382-bp fragment of the *gltA* gene [22]. Sterile water and DNA extracted from uninfected ticks from our laboratory were used as negative controls. Sequences obtained from PCR products were identified by comparison with GenBank [22]. All *gltA*-positive DNA samples were tested using multi-spacer typing [23]. For each sample, three intergenic spacers, (*dksA-xerC*, *mppA-purC* and *rpmE*-tRNAf^Met^) were amplified and sequenced using the primer pairs *dksA*F-*dksA*R, *mppA*F-*mppA*R and *rpmE*F-*rpmE*R, respectively. All amplicon sequences were compared to GenBank [22],[23].

### Epidemiological, entomological and climatic investigations

**Figure 2:**
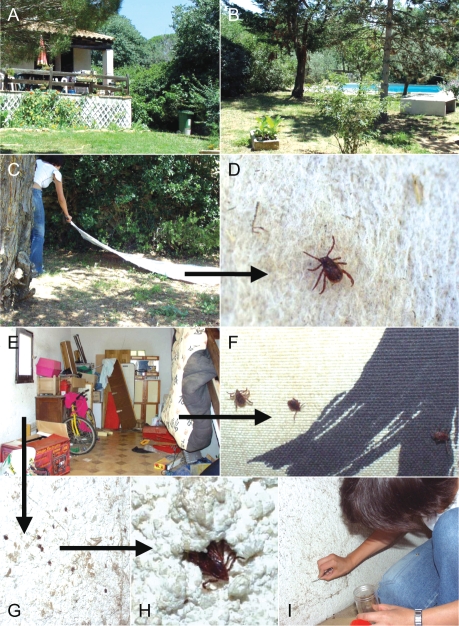
Entomological survey in the homesites where two patients where infected by *Rickettsia conorii* and *R. massiliae*. Panels A and B show the house and the garden where the patients were bitten by ticks. Panel C shows the collection of ticks by “flagging” or “dragging” a blanket over vegetation (Panel C). Ticks become attached to the blanket and can be removed periodically (Panel D). Panel E shows the garage where a dog used to sleep. Many *Rhipicephalus sanguineus* were found in crevices and cracks in the wall (Panel G and H) and on a blanket (F) and were collected (panel I) for molecular detection of rickettsias.

On July 5^th^ 2007, the house where the patients reported to have been bitten by ticks was visited. The owners were interviewed. Ticks were collected from walls of the house and from the garden (**Figure 2**) and were morphologically identified [24]. The climatic conditions in Nîmes in April and May 2007 were studied using the Météo-France's web site (http://www.meteo.fr/meteonet_en/index.htm).

### Testing the affinity of *Rh. sanguineus* for biting humans

We used larvae, nymphs and adults from a pathogen-free laboratory colony of *Rh. sanguineus*, that were colonized starting August 2006 when engorged *Rh. sanguineus* females were collected in Oran, Algeria, and maintained in environmental incubators at 25°C and 90% RH with a day/night photoperiod of 16∶8 (L∶D) h until they oviposited. Eggs and all life-cycle stages of subsequent generation were maintained under the same environmental conditions. For their blood meal, all stages were placed on a rabbit to feed until repletion [25]. The third generation of ticks was used for the experiment. For all stages, two batches were put on the arm of 3 human volunteers (3 of the authors including PP, IB, DR) who gave written consent to participate. This healthy volunteer study was approved by the Ethical Review Committee of the Faculty of Pharmacy, Algiers, Algeria. One batch was maintained the night before the test 25°C, and the other was maintained at 40°C. All ticks from the different groups were stored in environmental incubators with 90% relative humidity. All ticks were removed after 40 minutes and the number of attached ticks was compared between conditions (Mantel-Haenszel test). Three experiments were processed for larvae and nymphs, two for adults. An additional experiment was made that compared in the same experimental design, the affinity for biting of nymphs maintained at 32°C and 25°C.

## Results

### Diagnosis of rickettsioses in patients

**Figure 3:**
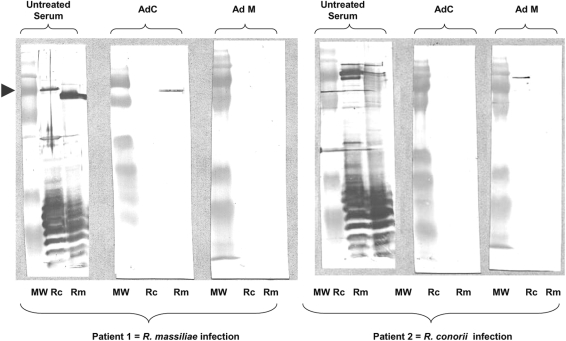
Western blot assay (WB) and cross adsorption studies of 2 patients with severe tick-borne rickettsioses in Nîmes, southern France, 2007. WB procedures were performed as described elsewhere [47] using 20 µl of a 1 mg/ml suspension of rickettsial antigen per lane. The cross-adsorption assay using *R. massiliae* and *R. conorii* antigens followed by WB on the resulting supernatant was performed as previously described [47]. Columns Rc and Rm depict western blots using *R. conorii* and *R. massiliae* antigens, respectively. Molecular weights (MW) are indicated on the left (arrow = 135 kDa). Untreated sera is acute sera tested by WB. For patient 1, when adsorption is performed with *R. massiliae* antigens (columns AdM), it results in the disappearance of homologous and heterologous antibodies. In contrast, when absorption is performed with *R. conorii* antigens (columns AdC), only homologous antibodies disappear indicating that antibodies are specific for *R. massiliae*. For patient 2, when adsorption is performed with *R. conorii* antigens (columns AdC), it results in the disappearance of homologous and heterologous antibodies. However, when it is performed with *R. massiliae* antigens (columns AdM), only homologous antibodies disappear indicating that antibodies are specific for *R. conorii*.

Using IF, antibodies against all spotted fever group rickettsial antigens were detected in patient 1 at the same level (IgG 2,048, IgM 16, on July 8^th^). In patient 2, the difference in titers between several antigens was lower than two-fold (IgG 1,024, IgM 256 for *R. conorii* ; IgG 1,024, IgM 128, for all other antigens). Western blot and cross-adsorption assays indicated that antibodies were specifically directed against *R. massiliae* in patient 1 and *R. conorii* in patient 2 (**Figure 3**). “Suicide PCR” was positive from two samples (of seven tested) obtained from the eye acqueous humor and the eschar biopsy of patient 1. Amplicon sequencing confirmed that patient 1 was infected with *R. massiliae*, as the obtained *pgsA* sequence was 98.9% similar to *R. massiliae*.

### Epidemiological and entomological investigation in patients' home sites

The owner, a 50 year-old nurse of the house were patient 1 and patient 2 had been bitten by tick was interviewed. Her dog that used to roam freely and used to rest and sleep in the garage and many rooms of the house, died due to a gastric torsion in April 2007. She reported that the ticks on this dog were numerous and particularly aggressive to people in April 2007, including before its death. The ticks regularly bit her, and her son, a 30 year-old man (not tested here) who presented at that time with a febrile syndrome and a maculo-papular rash. He did not sick medical care. However, when a veterinary doctor was consulted about the dog, he suggested that the son could have a “boutonneuse fever”, the other name of Mediteranean spotted fever, and suggested a doxycycline treatment. Fever disappeared on day 2 of a 200 mg daily doxycycline treatment and the son remained well.

The case-patients reported tick bites when they were outside, although they also noticed ticks in several walls inside the house. The entomological investigation was performed after the patients had cleared of brushwood and sprayed the garden with acaricides. A total of 218 nonengorged ticks, all identified as adult *Rh. sanguineus*, were collected in less than one hour. Ten ticks were collected with flannel flags dragged over vegetation. In the garage, 25 ticks were recovered from a blanket and 90 were collected from the walls inside (**Figure 2**). Outside, 93 ticks were collected from the walls of the house.

### Identification and genotyping of rickettsiae detected in ticks

Rickettsial DNA was detected in 37/133 ticks tested by PCR (28%). Twenty-four (18%) exhibited a 100% sequence similarity to *R. conorii* subsp. *conorii* strain Malish (AE008677). Thirteen specimens (10%) showed 100% sequence similarity with *R. massiliae* (U59720). Each of the *dks*A-*xer*C, *mpp*A-*pur*C, and *rpm*E-tRNA^fMet^ intergenic spacers were amplified from *gltA*-positive ticks. For all 24 *R. conorii*-positive ticks, *mpp*A-*pur*C sequences were 100% similar to *R. conorii* genotype A (AY345089), and *rpmE*-tRNA^fMet^ sequences were 100% similar to *R. conorii* genotype B (AY345092), but *dks*A-*xer*C sequences represented a new genotype named AX (EU081773). For all 13 *R. massiliae*-positive ticks, *dks*A-*xer*C sequences were 100% similar to *R. massiliae* genotype AE (CP000683), *rpm*E-tRNA^fMet^ sequences were new (genotype AD, EU250277), and *mppA-purC* sequences were also new (genotype AH, EU250278). All together, the two rickettsia represented new genotypes.

### Climatic conditions

**Figure 4:**
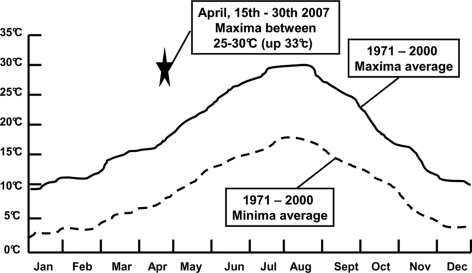
Monthly mean temperature (minimal and maximal) averaged from 1971 to 2000 in Nîmes southern France. The star symbolizes April 2007 which was the warmest April since 1950 when the focus of infection from an attack of *Rh. sanguineus* ticks was investigated (modified from [26]).

In April 2007, the weather in southern France was associated with the highest temperatures noted since 1950 (+3 to +4°C compared to seasonal norms) [26], particularly in the Gard region (Nimes being the main town). After April 15^th^, in Nîmes, maximal temperatures were continuously between 25°C and 30°C (**Figure 4**). A total of 15 “warm days” (>25°C) were recorded, in contrast to the seasonal norm of 0.6. The total duration of sunshine during the month was also increased when compared to seasonal norms (260h14min; that is+40 h). Few periods of rainfall were recorded between December 2006 and April 2007, with a total of 114.4 mm during this period, making this the 4^th^ dryest since 1921. In May, new records were reached with temperatures higher than 33°C between the 22^nd^ and 24^th^ [26].

### Affinity of *Rh. sanguineus* for biting humans

**Figure 5:**
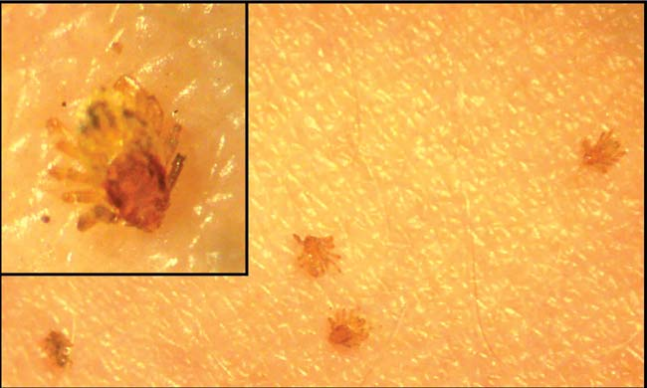
*Rhipicephalus sanguineus* nymphs found attached to human skin 40 min after having been placed on the arm. These ticks had been maintained at 40°C for one night before being placed on the arm.

In the 3 experiments with larvae, 27–67% of the ticks previously maintained at 40°C attached to the skin, whereas 0–6% of those at room temperature attached (p<0.05 in all cases). Among the nymphs, 10–20% of the batches previously maintained at 40°C attached to the skin (**Figure 5**), whereas none of those at room temperature attached. Overall, for larvae and nymphs, the number of ticks attached to the skin was dramatically higher for the group maintained at 40°C (**Table 1**). No difference appeared in adults, as no specimen but one attached to the skin within 40 mn. In the fourth additional experiment the affinity for biting of nymphs maintained at 32°C was also significantly higher than at 25°C (**Table 1**).

**Table 1:** Pathogen-free laboratory *Rhipicephalus sanguineus* put on the arm of a human volunteer.

Ticks stages/température	Number of tick tested by experiment	Number of tick found attached after 40 minutes (%)
Larvae/25°C		
Exp. 1	30	2 (6.7%)^a^
Exp. 2	60	0^b^
Exp. 3	30	0^c^
Larvae/40°C		
Exp. 1	30	8 (27%)^a^
Exp. 2	60	37 (66%)^b^
Exp. 3	30	17 (57%)^c^
Nymphs/25°C		
Exp. 1	30	0^d^
Exp. 2	10	0
Exp. 3	30	0^e^
Exp. 4	30	1 (3.3%)
Nymphs/40°C		
Exp. 1	30	6 (20%)^d^
Exp. 2	10	1
Exp. 3	30	4 (13.3%)^e^
Nymphs/32°C		
Exp. 4	30	9 (30%)
Adults/25°C		
Exp. 1	20	0
Exp. 2	30	0
Adults/40°C		
Exp. 1	30	0
Exp. 2	20	1

One batch of each stage of 3 experiments was maintained the night before the test at room temperature (25°C), and the other was maintained at 40°C. All ticks were removed after 40 minutes.

Experiments 1 to 3 were performed using larvae and nymphs, 2 weeks old ticks. Experiment 4 was performed using 2 months old nymphs. ^a, b, c, d, e^ : p<0.05 (Mantel-Haenszel test).

## Discussion

When investigating these grouped cases of severe spotted fevers first presumed to be MSF caused by *R. conorii*, two rickettsial pathogens were in fact identified. This report describes the second human case of *R. massiliae* infection and was documented using the IF reference serology assays [5], completed by western blot and cross absorption studies and definitely confirmed with the use of molecular tools. *R. massiliae* is a worldwide rickettsia that was isolated in 1992 and thereafter detected in *Rhipicephalus* spp. in Europe and Africa [5], Argentina [27], and recently in Arizona, USA [15]. The recognition of the pathogenicity of *R. massiliae* occurred in 2005 when molecular tools were used to identify a rickettsial isolate obtained 20 years before from a man hospitalised in Italy with fever, an eschar, and a maculopapular rash [16]. In fact, *R. massiliae* is the sole pathogenic rickettsia known to be prevalent in America, Africa and Europe. In the present report, the predominant symptom was acute visual loss, and both diagnosis and treatment were delayed. Although eye involvement has been reported in spotted fever group rickettsioses, these manifestations are underdiagnosed or frequently misdiagnosed [28]–[30]. Clinicians should suspect rickettsioses in patients with febrile acute visual loss, particularly during the warmest and most common months for *Rh. sanguineus*-transmitted diseases.

Indeed, we have identified here that the source of the focus was an unexpected attack of *Rh. sanguineus*. Additionally, we have shown for the first time that the population of rickettsias found in a focus of infection was clonal, as all *R. massiliae*- and *R. conorii*-positive samples had a unique MST genotype. The rate of infection of *Rh. sanguineus* was high, particularly for *R. conorii* (18%). In contrast, the rate of infection is usually lower that 1% [5],[31]. This, combined with an unusual rate of tick attack, was responsible for multiple inoculation escar in our patients. This is an unusual finding in most tick-borne rickettsial diseases, including MSF, because the probability of being bitten simultaneously by several infected ticks has been considered to be rare. This finding is characteristic of few other tick-borne rickettsioses, an example being African tick bite fever caused by *R. africae*, due to the aggressive behavior of the tick vectors and a high tick infection rate [5],[32].

*Rh. sanguineus* lives in peridomestic environments shared with dogs but is known to have a low affinity for humans. Hosts other than dogs are usually only infested when dogs are present to maintain a population of the tick [33]. In this setting, the dog allowed a large infestation of ticks, which had no place to go when the dog died. The risk of the people to be bitten was therefore greatly increased, very much like relapsing fever in the American West, where infected soft ticks accumulate in cabins when their squirrel hosts die during the winter [34]. This highlights the importance of the so-called “zooprophylaxis” – that risk is small when there are alternative hosts than people upon which a vector can focus. However, in the present report, ticks started to be particularly aggressive to people before the death of the dog. Also, the son of the owner presented with a tick-borne eruptive fever, before the dog died. Therefore, the death of the dog could not be considered as the cause of this unusual cluster of *Rh. sanguineus* transmitted rickettsioses.We provide some evidence that this cluster of cases was related to unusually warm temperatures. As shown herein, April 2007 was the warmest April since 1950, with summer-like temperatures [26]. Also, considerable evidence was accumulated by investigators in the 1940's about the role of *Rh. sanguineus* as a vector of rickettsioses in warm countries such as Mexico [5]. In Europe and North Africa, although *Rh. sanguineus* starts to be active in May and June [24], most cases of MSF are diagnosed during July and August. This is probably linked to an increased aggressiveness and propensity of *Rh. sanguineus* to bite hosts in warmer conditions, as demonstrated for other *Rhipicephalus* species biting cattle [35],[36]. During the 1970s, the increase in the number of MSF cases observed in southern Europe [37] was correlated with higher temperatures and lower rainfall in Spain, and with a decrease in the number of days of frost during the preceding year in France [38],[39]. The cases of MSF recognized in Oran, Algeria in 1993 peaked in 2005 together with the hottest summer of the past decades [18]. More recently, maximum temperature levels during the previous summer were associated with increases in MSF incidence in Sardinia [40]. Finally, during the French heat wave in 2003, with the hottest summer of the preceding 50 years, 22 *Rh. sanguineus*, including specimens infected by *R. conorii* and *R. massiliae*, were found attached to an homeless person who died of MSF in August [41]. This case was highly unusual in regard to the intensity of the parasitism by *Rh. sanguineus* which had never been reported before in patients or by entomologic investigators who spent their lives collecting ticks [38].

Herein, we have demonstrated by our experimental model that the aggressiveness of immature stages of *Rh. sanguineus* to bite human is modulated by external temperature. It is important to remind that ixodids do not cause pain while feeding and immature stages are frequently not detected on people because of their small size [6]. Furthermore, we have recently demonstrated the similar effects of higher temperature on the speed of attachment of all stages of *Rh. sanguineus* ticks, including adults using a rabbit model in a similar experimental design and a 48 hour observation period [42]. We conclude that the host seeking and feeding behaviors of *Rh. sanguineus* in the present focus were modified by the warmer climatic circumstances and became highly aggressive for the owners and visitors of the house.

*Rh. sanguineus*, a tick of African origin, is now of global importance [11]. The public health importance of the globalisation of vector-borne diseases has been illustrated with West-Nile fever that emerged in 1999 in the USA and has become the dominant vector-borne viral disease [43]. More recently, a returned traveller served as a source of a local Chikungunya virus outbreak in Europe [2], where *Aedes albopictus*, the recently globalised Asian tiger mosquito is prevalent, as it also is in America [44]. Although ticks have long been considered vectors of geographic diseases because of their preferred environmental conditions and biotopes [6], some vectors have also been globalised. *R. africae*, the agent of African tick bite fever, has been found in the West Indies where it was introduced from Africa during the 18th century through *Amblyomma variegatum* ticks on cattle. Now, this rickettsia threatens the American mainland [45].

**Figure 6:**
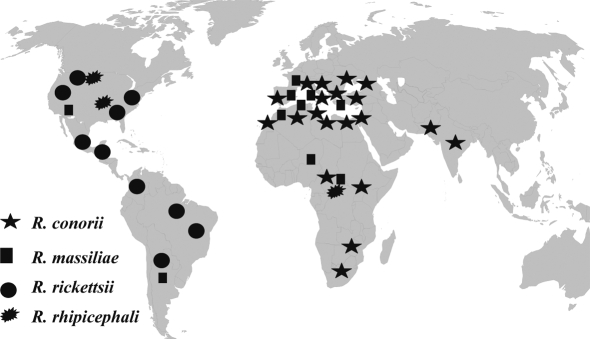
Global distribution of spotted fever group rickettsias potentially transmitted by *Rh. sanguineus*, threatening humans all over the world, including 3 recognized pathogens *R. conorii*, *R. massiliae*, *R. rickettsii* (which is also transmitted by *Dermacentor* spp, and *Amblyomma* spp), and *R. rhipicephali*, a rickettsia of unknown pathogenicity.

*Rh. sanguineus* has spread globally between 50°N and 35°S because of its ability to survive in human home sites [11]. Climate variability will change local weather in sites where brown dog tick infestations occur. If global trends in weather over the long term unfold as predicted, weather will be more variable and may comprise warmer temperatures, droughts and heat waves, as well as more monsoons, or more intense snowstorms, depending on the site [9],[46]. Based on the present investigation and previous epidemiological clinical and experimental data (**Table 2**), and on a global perspective, we predict that increased temperature will lead to an increased period of activity of *Rh. sanguineus* and an increased aggressiveness and proclivity to bite humans, and that increased incidence of *Rh. sanguineus*-transmitted diseases will be observed (**Figure 6**). On a flip side, cooler weather, if any, in sites where *Rh. sanguineus* are currently endemic would imply less human biting. However, in the context of warming [9], the public health burden of this tick will increase. After *R. conorii*, *R. rickettsii* and the worldwide emerging pathogen *R. massiliae* [5], other rickettsial agents such as *R. rhipicephali* [5], or yet undescribed microorganisms, could be found soon as emerging pathogens transmitted by the globalised and multipotent vector, *Rh. sanguineus*.

**Table 2:** Evidence of the influence of warmer weather and climate on *Rh. sanguineus* transmitted rickettsioses in humans.

**Epidemiological evidence**
• France, this report. Unsual cluster of cases in an atypical period of the year: April 2007 was the warmest April since 1950, with summer-like temperature
• Southern USA, and Central America: role of *Rh. sanguineus* as a vector of *R. rickettsii* in warm states (Arizona) [14] or countries (Mexico) [5].
• Europe and North Africa: *Rh. sanguineus* starts to be active in May and June [24], but most cases of MSF are diagnosed during the warmest months, July and August.
• Southern Europe, the 1970s: the increase in the number of MSF cases [37] was correlated with higher temperatures and lower rainfall in Spain, and with a decrease in the number of days of frost during the preceding year in France [39].
• Oran, Algeria: the cases of MSF peaked in 2005 together with the hottest summer of the past decades [18].
• Sardinia, Italy: maximum temperature levels associated with increases in MSF incidence in Sardinia [40].
**Clinical evidence**
• France, French heat wave in 2003: hottest summer of the preceding 50 years, 22 *Rh. sanguineus*, including specimens infected by *R. conorii* and *R. massiliae*, were found attached to an homeless person, who died of MSF [41].
• This report: multiple escars unusual finding in MSF, because the probability of being bitten simultaneously by several infected *Rh. sanguineus* ticks is considered to be rare.
• Multiple eschars in MSF reported in the warmest countries of southern Europe (Spain) [48].
**Experimental models**
• Increased aggressiveness and propensity of *Rh. sanguineus* to bite unusual hosts (rabbit) in warmer conditions [42]
• This study: Increased aggressiveness and propensity of *Rh. sanguineus* to bite hosts in warmer conditions
